# Magnetic Micro Sensors with Two Magnetic Field Effect Transistors Fabricated Using the Commercial Complementary Metal Oxide Semiconductor Process

**DOI:** 10.3390/s20174731

**Published:** 2020-08-21

**Authors:** Wei-Ren Chen, Yao-Chuan Tsai, Po-Jen Shih, Cheng-Chih Hsu, Ching-Liang Dai

**Affiliations:** 1 Department of Mechanical Engineering, National Chung Hsing University, Taichung 402, Taiwan; wei-then@hotmail.com; 2 Department of Bio-Industrial Mechatronics Engineering, National Chung Hsing University, Taichung 402, Taiwan; yctsaii@dragon.nchu.edu.tw; 3 Department of Biomedical Engineering, National Taiwan University, Taipei 106, Taiwan; pjshih@ntu.edu.tw; 4 Department of Electro-Optical Engineering, National United University, Miaoli 360, Taiwan; cchsu920624@nuu.edu.tw

**Keywords:** magnetic microsensor, magnetic field effect transistor, CMOS, MEMS

## Abstract

The fabrication and characterization of a magnetic micro sensor (MMS) with two magnetic field effect transistors (MAGFETs) based on the commercial complementary metal oxide semiconductor (CMOS) process are investigated. The magnetic micro sensor is a three-axis sensing type. The structure of the magnetic microsensor is composed of an x/y-MAGFET and a z-MAGFET. The x/y-MAGFET is employed to sense the magnetic field (MF) in the *x*- and *y*-axis, and the z-MAGFET is used to detect the MF in the *z*-axis. To increase the sensitivity of the magnetic microsensor, gates are introduced into the two MAGFETs. The sensing current of the MAGFET enhances when a bias voltage is applied to the gates. The finite element method software Sentaurus TCAD was used to analyze the MMS’s performance. Experiments show that the MMS has a sensitivity of 182 mV/T in the *x*-axis MF and a sensitivity of 180 mV/T in the *y*-axis MF. The sensitivity of the MMS is 27.8 mV/T in the *z*-axis MF.

## 1. Introduction

Magnetic micro sensors (MMS) play an important role in measurement of magnetic field and are applied in various fields. For instance, Mohri [1] employed amorphous wire complementary metal oxide semiconductor (CMOS) integrated circuit (IC) magnetoimpedance to design a magnetic micro sensor, and the sensor was applied in the electric compass of smartphones and the magnetic guidance self-driving system. A respiratory training and monitoring system for radiotherapy, presented by Oh [2], was developed using a microelectromechanical system (MEMS) magnetic micro sensor (MMS). This system was composed of an MEMS MMS, a small magnet and a breathing output component. Sideris [3] has proposed a 2 × 2 MMS fabricated using the CMOS technology. The MMS array was applied in bio-detection assays. A three-axis MEMS MMS, fabricated by Li [4], was a digital tunneling magnetoresistance type. The MMS with a CMOS interface circuit has been used in the field of nanosatellites. Vetrella [5] has used an MEMS MMS to design a cooperative unmanned aerial vehicle navigation system. The system includes an MMS, an inertial sensor, a global positioning system receiver, a vision component, and a navigation algorithm to stabilize and control the flight of an unmanned aerial vehicle. A tunnel magnetoresistance MMS, designed by Tavassolizadeh [6], has been applied in sensing micro-and nano-scale strain. The experiments showed that the tunnel magnetoresistance MMS has an ability to measure both compressive and tensile stresses. Gooneratne [7] has made a micro-chip consisting of a magnetoresistive MMS and a unique magnetic actuator. The micro-chip can integrate with microfluidic components and electronic circuitry for biomolecule quantification detection. Zhang [8] has used a three-axis MMS to measure the steel condition in reinforced concrete bridges. Jogschies [9] has introduced a magnetoresistive MMS manufactured on a flexible substrate. The flexible MMS has been applied to the flexible write head of data storage components.

In addition to the manufacture of integrated circuits, the standard CMOS process is also used to produce various micro sensors [10,11,12,13] and microactuators [14,15]. Many MMSs have also been developed using the standard CMOS process. For example, Li [16] developed an MMS with a conducting magnetic structure using a CMOS process. The MMS was bonded on a printed circuit board. The sensor became more sensitive due to the conducting magnetic structure located above the MMS. The MMS sensitivity was 132 mV/T. Oh [17] used a CMOS process to design a vertical-type Hall MMS with a four-contact structure. Compared with Oh’s previous MMS [17], the sensitivity of the sensor increased by 13 times because of the vertical-type design and four-contact structure. A three-axis MMS, presented by Lin [18], was made using CMOS technology. The MMS consisted of four Hall elements and a magnetotransistor. The four Hall elements detected the magnetic field (MF) in *z*-axis. The magnetotransistor measured the magnetic field in the *x*/*y*-axis. The MMS had a sensitivity of 0.69 V/T in the *x*-axis MF and a sensitivity of 0.55 V/T in the *y*-axis MF. The sensitivity of the MMS exceeded that of Li [16]. Osberger [19] presented an MMS fabricated using a standard CMOS process. A chopper-stabilized magnetic field effect transistor was employed to design the MMS and to enhance the sensing resolution of the MMS. A three-axis MMS, proposed by Tseng [20], was made using a standard CMOS process. The MMS was a magnetotransistor that included eight collectors, four bases, and a ring emitter. Amplifier circuitry integrated with the MMS magnified the voltage output of the MMS. The experimental results showed that the MMS had a sensitivity of 6.5 mV/T in the *x*-axis MF and a sensitivity of 6.5 mV/T in the *y*-axis MF. The sensitivity of the MMS was lower than that of Li [16] and Lin [18]. A fluxgate MMS, which was presented by Lu [21], was produced using CMOS-MEMS technology. The fluxgate MMS was bounded on a printed circuit board using a flip-chip packaging method, and the printed circuit board had planar pick-up coils, three-dimensional excitation coils, and dual magnetic cores. The maximum responsivity of the fluxgate MMS was 593 V/T at the excitation frequency of 50 kHz. The minimum MF noise of the fluxgate MMS was 0.05 nT/$\sqrt{\mathrm{Hz}}$. The fluxgate MMS required a post-CMOS process; thus, the fabrication of the fluxgate was more complicated than that of Li [16], Oh [17], Lin [18], Osberger [19], and Tseng [20]. A three-axis MMS, developed by Leepattarapongpan [22], was made using a CMOS process. The MMS was a magnetotransistor, its structure including four collectors, four bases, and one emitter. The MMS sensitivity was 14.5%/T in the range 0–400 mT. Sung [23] fabricated an MMS using a CMOS process. The MMS was a folded vertical Hall element. The MMS used a lateral folded structure and a guard ring to reduce the cross-coupling noise. Kimura [24] developed a two-dimensional MMS using a standard CMOS process. The MMS consisted of a 64 × 64 array of the Hall element, and it had a low-frequency noise of 16%. Jiang [25] utilized a standard CMOS process to make an MMS. The MMS that combined spinning-current Hall elements and pick-up coils had a resolution of 210 μT in a bandwidth of 3 MHz. A two-dimensional MMS, proposed by Kimura [26], was made based on the CMOS technology. The MMS was a 16 × 16 array of the Hall element, and the average sensitivity of the MMS was 0.140 mV/mT. The sensitivity of MMS approached that of Li [16] but was smaller than that of Lin [18].

These magnetic micro sensors [16,17,18,19,21,23,25,26] fabricated by CMOS technology were 1-axis MF sensors. The MMS developed by Lin [18] was a three-axis sensor, but its cross-sensitivity was high because the structure of the MMS had only one magnetotransistor. Therefore, this work develops a three-axis MMS fabricated by the commercial CMOS process. To avoid the cross-coupling effect and reduce the cross-sensitivity, the MMS was composed of two magnetic field effect transistors (MAGFETs): an x/y-MAGFET and a z-MAGFET. The x/y-MAGFET measured the MF in the *x*- and *y*-axis, and the z-MAGFET measured the MF in the *z*-axis.

## 2. Structure of the MMS

**Figure 1:**
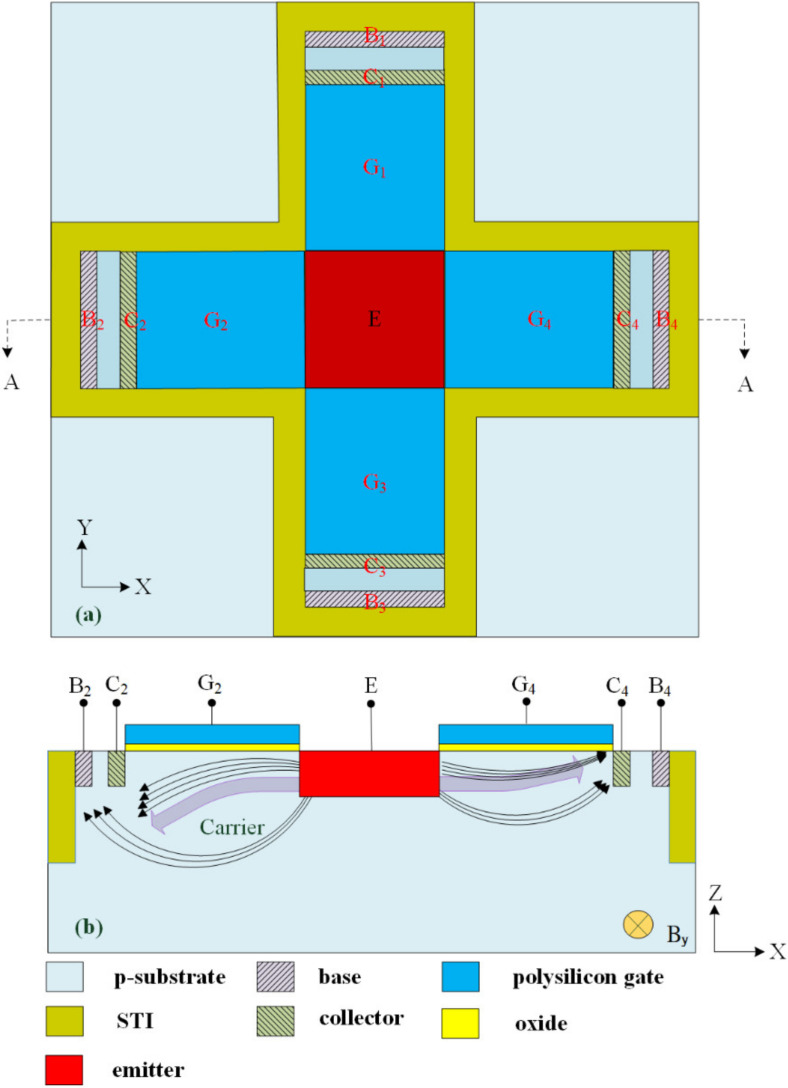
(**a**) Structure of the x/y magnetic field effect transistor (MAGFET); (**b**) Cross-sectional view of the x/y-MAGFET along line AA.

The magnetic micro sensor is composed of an x/y-MAGFET and a z-MAGFET. The x/y-MAGFET detects the MF in the *x*- and *y*-axis. The z-MAGFET senses the MF in the *z*-axis. Figure 1a illustrates the structure of the x/y-MAGFET, where B1, B2, B3, and B4 are the bases of the x/y-MAGFET; C1, C2, C3, and C4 are the collectors of the x/y-MAGFET; G1, G2, G3, and G4 are the gates of the x/y-MAGFET; and E is the emitter of the x/y-MAGFET. The x/y-MAGFET uses shallow trench isolation (STI) oxide to limit the current movement direction and to reduce the leakage current.

Figure 1b illustrates a cross-sectional view of the x/y-MAGFET along line AA. When a bias voltage is applied to the gates of the x/y-MAGFET, the surface of the p-substrate forms a channel under the gate, increasing the mobility of carriers and enhancing the sensitivity of the MMS. The sensing principle of the x/y-MAGFET is as follows. Carriers move from the emitter to the bases and collectors through applying the bias voltages to the bases, collectors, and gates. Because the Lorentz force acts, carriers on the right in Figure 1b are deflected upward when an MF in the *y*-axis applies to the x/y-MAGFET. A small number of carriers move to the base B_4_ owing to the collector C_4_ obstructing their movement paths. Most carriers move to the collector C_4_, resulting in the current of the collector C_4_ increasing. On the other hand, because the Lorentz force acts, carriers on the left in Figure 1b are deflected downward when an MF in the *y*-axis applies to the x/y-MAGFET. Most carriers pass across the collector C_2_ and move to the base B_2_, resulting in the current of the collector C_2_ decreasing. Thereby, the x/y-MAGFET has a voltage difference between the collectors C_2_ and C_4_ in the *y*-axis MF. The voltage difference of the collectors C_2_/C_4_ is the output voltage (OV) of the MMS in the *y*-axis MF.

Similarly, carriers move from the emitter to the bases and collectors through applying the bias voltages to the bases, collectors, and gates. Because the Lorentz force acts, carriers moving to the collector C_1_ are deflected downward when an MF in the *x*-axis is applied to the x/y-MAGFET. Most carriers pass across the collector C_1_ and move to the base B_1_, resulting in the current of the collector C_1_ decreasing. Additionally, because the Lorentz force acts, carriers moving to the collector C_3_ are deflected upward when an MF in the *x*-axis is applied to the x/y-MAGFET. A small number of carriers move to the base B_3_ owing to the collector C_3_ obstructing their movement paths. Most carriers move to the collector C_3_, increasing the current of the collector C_3_. Thereby, the x/y-MAGFET has a voltage difference between the collectors C_1_ and C_3_ in the *x*-axis MF. The voltage difference of the collectors C_1_/C_3_ is the MMS OV in the *y*-axis MF.

**Figure 2:**
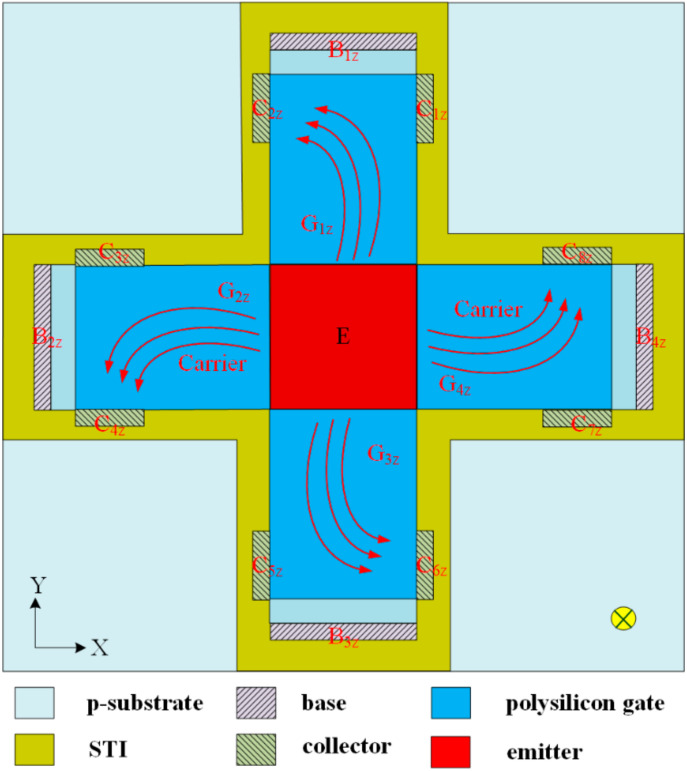
Structure of the z-MAGFET.

Figure 2 illustrates the structure of the z-MAGFET, where B_1z_, B_2z_, B_3z_, and B_4z_ are the bases of the z-MAGFET; G_1z_, G_2z_, G_3z_, and G_4z_ are the gates of the z-MAGFET; E_z_ is the emitter of the z-MAGFET; and C_1z_, C_2z_, C_3z_, C_4z,_ C_5z_, C_6z_, C_7z_, and C_8z_ are the collectors of the z-MAGFET. The STI oxide limits the current movement direction and reduces the leakage current for the z-MAGFET. Carriers move from the emitter to the bases and collectors when bias voltages are applied to the gates, collectors, and bases. Because of the Lorentz force acting, carriers are deflected toward the collectors C_2z_, C_4z_, C_6z_, and C_8z_ when an MF in the *z*-axis is applied to the z-MAGFET. The current produces an imbalance between the collectors C_1z_ and C_2z_, resulting in the z-MAGFET having a voltage difference with the collectors C_1z_/C_2z_ in the *z*-axis MF. Similarly, the collectors between the electrodes C_3z_/C_4z_, C_5z_/C_6z_, and C_7z_/C_8z_, respectively, produce a voltage difference in the *z*-axis MF. All voltage differences in the series are the MMS OV in the *z*-axis MF.

**Figure 3:**
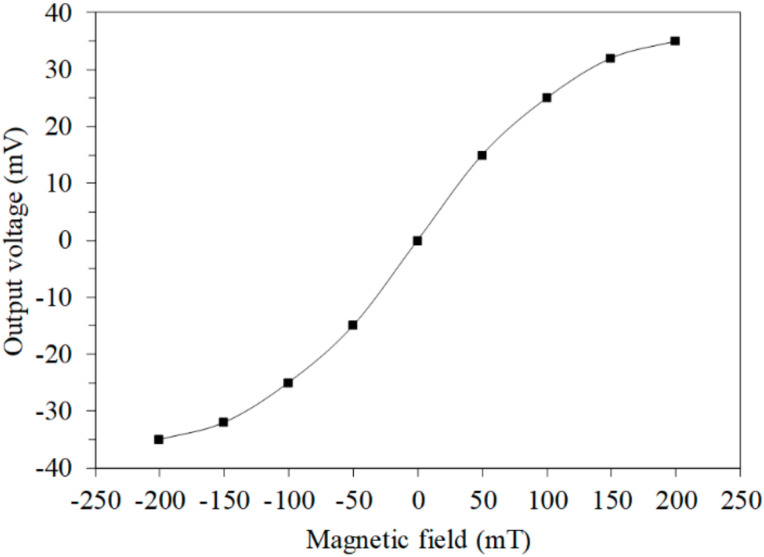
Simulated output voltage (OV) in the *y*-axis magnetic field.

The finite element method software Sentaurus TCAD was used to analyze the MMS characteristic. A model of the x/y-MAGFET (Figure 1a) was established, and the model was meshed using the Delaunay triangulation approach. The electrical and MF coupling effect for the x/y MAGFET was analyzed using the Poisson electron hole method. The carrier density distribution of the x/y-MAGFET was solved using the Bank–Rose method. Figure 3 shows the simulated OV for the MMS in the *x*-axis MF. In the simulation, the voltage of the bases and collectors for the x/y-MAGFET was 3.3 V. The bases and collectors connected with a resistance of 1 kΩ, respectively. The voltage of the gates for the x/y-MAGFET was 0.4 V. An MF in the *x*-axis was provided to the x/y-MAGFET. The simulated results showed that the MMS OV in the *x*-axis MF changed from −35 mV at −200 mT to 35 mV at 200 mT. The x/y-MAGFET was a symmetrical structure, so the MMS characteristic in the *y*-axis MF was the same as that in the *x*-axis MF. Thereby, the simulated OV of the MMS in the *y*-axis MF was the same as that of the MMS in the *x*-axis MF.

**Figure 4:**
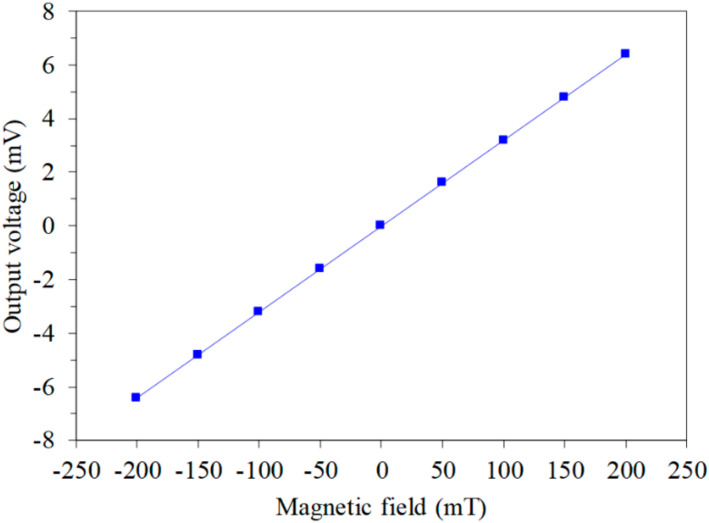
Simulated OV in the *z*-axis magnetic field.

With the same simulation method, the MMS OV in the *z*-axis MF was simulated. The model of the z-MAGFET (Figure 2) was established, and the OV of the z-MAGFET in the *z*-axis MF was simulated. Figure 4 shows the MMS OV in the *z*-axis MF. In the simulation, the voltage of the gates for the z-MAGFET was 0.4 V. The bases and collectors connected with a resistance of 1 kΩ, respectively. The voltage of the bases and collectors for the z-MAGFET was 3.3 V. The *z*-axis MF was applied to the z-MAGFET. The results showed that the simulated OV of the MMS in the *z*-axis MF varied from −6.5 mV at −200 mT to 6.5 mV at 200 mT.

## 3. Fabrication of the MMS

**Figure 5:**
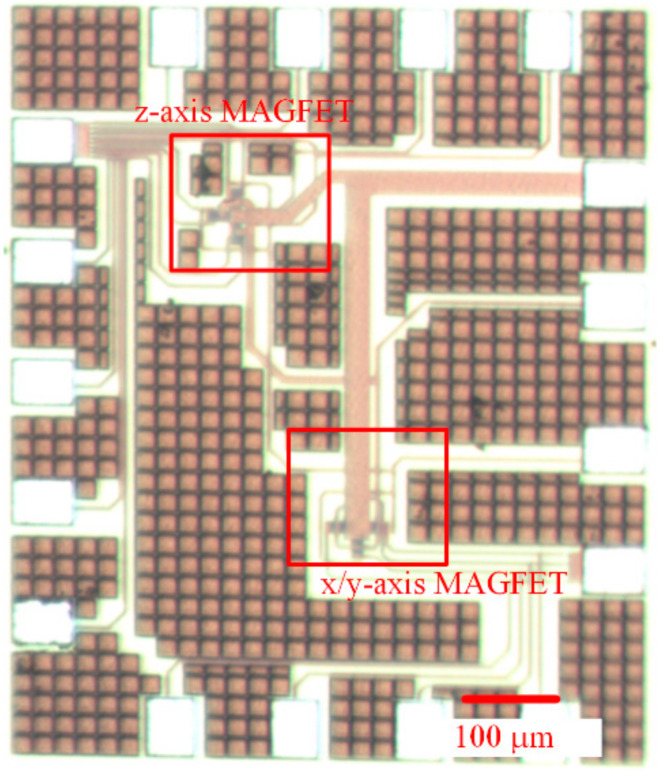
Optical image of the magnetic micro sensor (MMS) chip.

**Figure 6:**
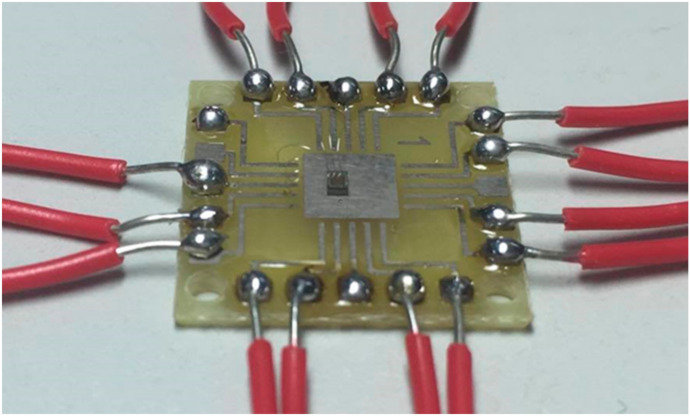
Picture of the MMS after wire-bonding.

The magnetic micro sensor consisted of an x/y-MAGFET and a z-MAGFET. Figure 1 shows the structure of the x/y-MAGFET, and Figure 2 shows the structure of the z-MAGFET. The layout of the x/y-MAGFET was designed according to the structure in Figure 1. At the same time, the layout of the z-MAGFET was designed in accordance with the structure in Figure 2. The MMS was manufactured utilizing the commercial CMOS process [27,28,29]. According to the layout of the MMS, the Taiwan Semiconductor Manufacturing Company (TSMC) made the MMS based on the commercial 0.18 μm CMOS process. Figure 5 demonstrates an optical image of the MMS after completion of the CMOS process. As shown in Figure 5, the MMS chip has a z-MAGFET and an x/y-MAGFET. To measure the characteristic of the MMS chip, the MMS chip was wire-bonded on a printed circuit board. Figure 6 shows a picture of the MMS after wire-bonding.

## 4. Results

**Figure 7:**
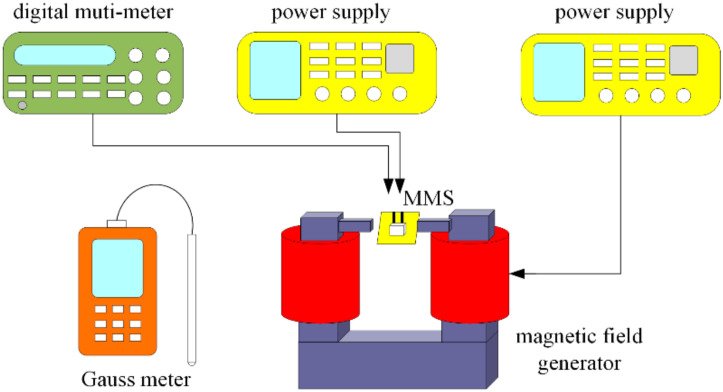
Experimental setup for the MMS.

Figure 7 shows the experimental setup of the MMS. A Gauss meter, a digital multimeter, a magnetic field generator (MFG), and two power supplies were employed to measure the MMS characteristic. The MMS was set in the MFG. One power supply provided power to the MFG, and the other one provided power to the MMS. The MFG produced an MF to the MMS. The magnitude of the MF generated by the MFG was calibrated using the Gauss meter. The MMS OV was recorded using the digital multimeter.

**Figure 8:**
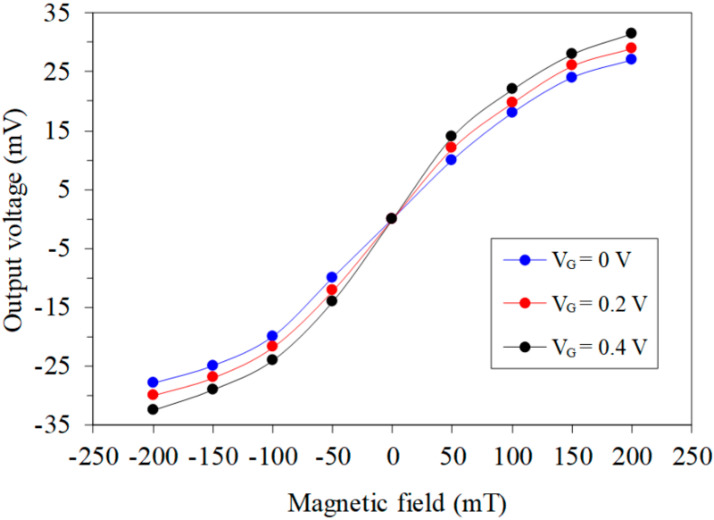
Tested OV in the *x*-axis magnetic field.

First, the characteristic of the MMS in the *x*-axis MF was measured. As shown in Figure 7, the MMS was set in the MFG, and the MFG provided an MF range of −200 to 200 mT to the x/y-MAGFET. The bases and collectors of the x/y-MAGFET were applied with a bias voltage of 3.3 V. The gates of the x/y-MAGFET were applied with different voltages. The bases and collectors connected with a resistance of 1 kΩ. The digital multimeter recorded the voltage difference of the collectors C_2_/C_4_ for the x/y-MAGFET. Figure 8 shows the tested OV for the MMS in the *x*-axis MF, where V_G_ is the gate voltage of the MAGFET. When V_G_ = 0 V, the MMS OV changed from −28 mV at −200 mT to 27.2 mT at 200 mT. When V_G_ = 0.2 V, the MMS OV varied from −29 mV at −200 mT to 30.3 mV at 200 mT. The MMS was more sensitive to the MF at V_G_ = 0.4 V, and its OV increased from −32.5 mV at −200 mT to 31.8 mV at 200 mT. The curves in Figure 8 are nonlinear. The least squares method was employed to analyze the linear regression of the curves. The analysis showed that the slope of the regression line at V_G_ = 0.4 V was 182 mV/T. The sensitivity of the MMS in the *x*-axis MF was 182 mV/T.

**Figure 9:**
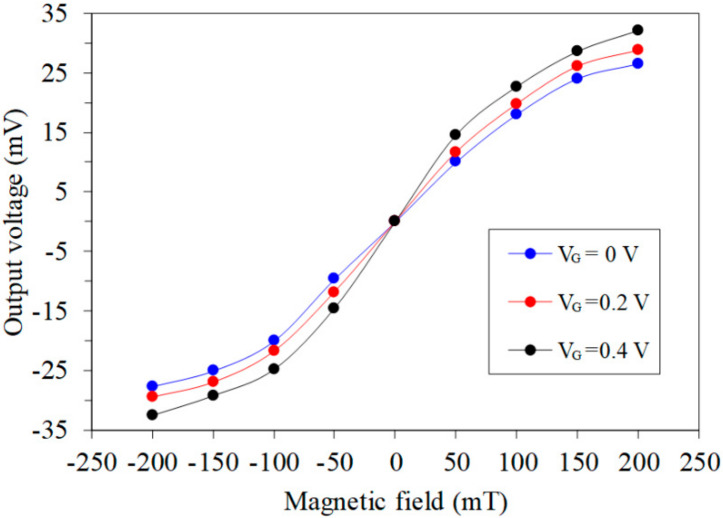
Tested OV in the *y*-axis magnetic field.

The characteristic of the MMS in the *y*-axis MF was tested. The MFG generated a *y*-axis MF and applied it to the x/y-MAGFET. The power supply provided a bias voltage of 3.3 V to the bases and collectors of the x/y-MAGFET. The gates of the x/y-MAGFET were applied with different voltages. The bases and collectors connected with a resistance of 1 kΩ. The voltage difference of the collectors C_1_/C_3_ for the x/y-MAGFET was measured using the digital multimeter. Figure 9 shows the tested OV for the MMS in the *y*-axis MF, where V_G_ is the gate voltage of the MAGFET. When V_G_ = 0 V, the sensitivity of the MMS was the lowest and its OV varied from −27.6 mV at −200 mT to 26.8 mT at 200 mT. When V_G_ = 0.2 V, the MMS OV changed from −29.5 mV at −200 mT to 28.7 mV at 200 mT. When V_G_ = 0.4 V, the MMS was more sensitive to the MF and its OV increased from −32.2 mV at −200 mT to 31.4 mV at 200 mT. The least squares method was utilized to evaluate the linear regression of the curves in Figure 9. The results showed that the slope of the regression line at V_G_ = 0.4 V was 180 mV/T. The sensitivity of the MMS in the *y*-axis MF was 180 mV/T.

**Figure 10:**
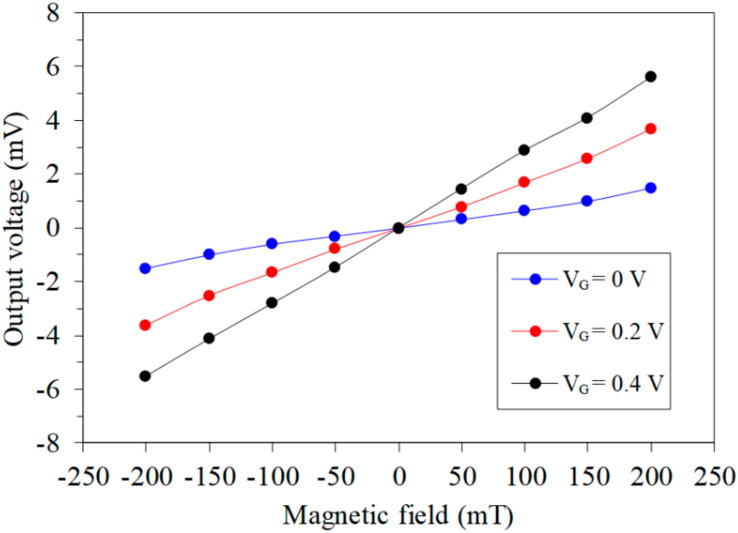
Tested OV in the *z*-axis magnetic field.

The characteristic of the MMS in the *z*-axis MF was tested. A *z*-axis MF, which was produced by the MFG, was applied to the z-MAGFET. A bias voltage of 3.3 V was applied to the bases and collectors of the z-MAGFET. The gates of the z-MAGFET were applied with different voltages. The bases and collectors connected with a resistance of 1 kΩ. The digital multimeter was used to test the OV of the z-MAGFET. Figure 10 shows the tested OV for the MMS in the *z*-axis MF, where V_G_ is the gate voltage of the MAGFET. The MMS was insensitive to the MF at V_G_ = 0 V. As the gate voltage increased, the MMS OV became strong. When V_G_ = 0.2 V, the MMS OV changed from −3.6 mV at −200 mT to 3.7 mV at 200 mT. When V_G_ = 0.4 V, the MMS OV increased from −5.6 mV at −200 mT to 5.8 mV at 200 mT. The slope of the curve at V_G_ = 0.4 V was 27.8 mV/T. Therefore, the sensitivity of the MMS in the *z*-axis MF was 27.8 mV/T. When the Lorentz force acts, the number of carriers (z-MAGFET in Figure 2) deflected to the side is smaller than the number of carriers (x/y-MAGFET in Figure 1) that are deflected upward or downward. Therefore, the output voltage and sensitivity of the z-MAGFET are smaller than that of the x/y-MAGFET.

**Figure 11:**
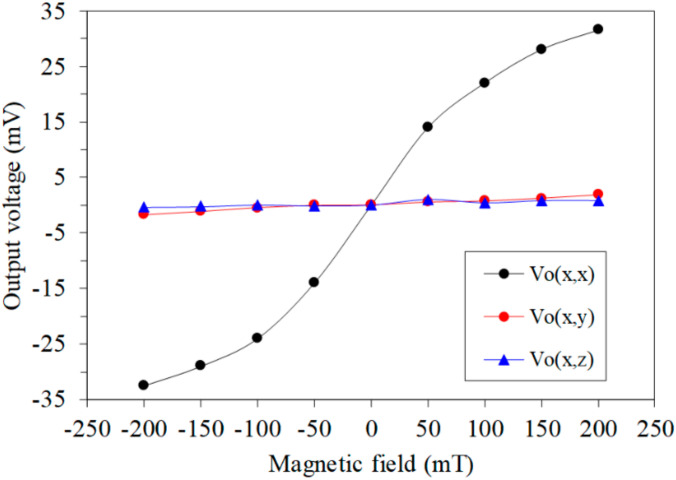
Output of the MMS in the *x*-axis magnetic field.

To understand the cross-sensitivity of the MMS, the OV for each axis of the MMS was measured in the same MF. First, an MF in the *x*-axis was applied to the MMS. The bases and collectors of the x/y-MAGFET and z-MAGFET were applied with a bias voltage of 3.3 V. A gate voltage of 0.4 V was applied to the gates of the x/y-MAGFET and z-MAGFET. The OVs of the x/y-MAGFET and z-MAGFET were measured using the digital multimeter. Figure 11 shows the OV for each axis of the MMS in the *x*-axis MF, where Vo(x,x) is the x-direction OV of the x/y-MAGFET in the *x*-axis MF; Vo(x,y) is the y-direction OV of the x/y-MAGFET in the *x*-axis MF; and Vo(x,z) is the OV of the z-MAGFET in the *x*-axis MF. As shown in Figure 11, the Vo(x,y) and Vo(x,z) are low in the *x*-axis MF. The slope of the curve Vo(x,y) was 8.2 mV/T, and the slope of the curve Vo(x,z) was 3.4 mV/T. Thereby, the MMS had a cross-sensitivity of 8.2 mV/T (y-direction output) and a cross-sensitivity of 3.4 mV/T (z-direction output) in the *x*-axis MF. The sensitivity of the MMS in the *x*-axis MF was 182 mV/T. Compared to the sensitivity of the MMS, the cross-sensitivity of the MMS in *x*-axis MF was less than 6%.

**Figure 12:**
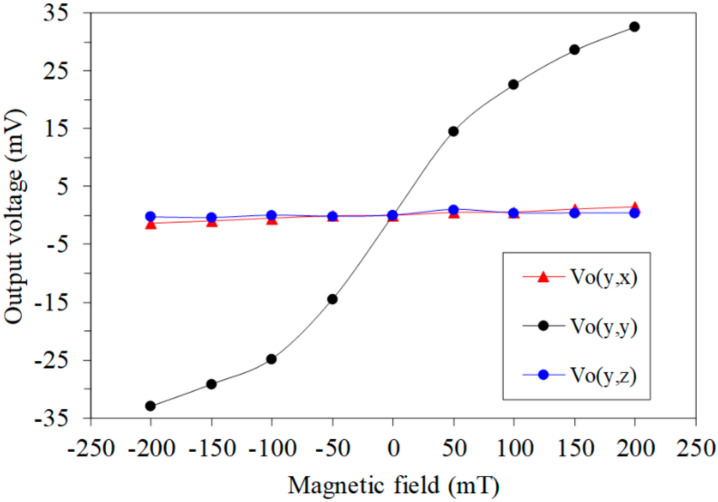
Output of the MMS in the *y*-axis magnetic field.

A magnetic field in the *y*-axis was applied to the MMS. A bias voltage of 3.3 V was applied to the bases and collectors of the x/y-MAGFET and z-MAGFET. The gate voltage of the x/y-MAGFET and z-MAGFET was 0.4 V. The digital multimeter recorded the OV of the x/y-MAGFET and z-MAGFET. Figure 12 shows the OV for each axis of the MMS in the *y*-axis MF, where Vo(y,x) is the x-direction OV of the x/y-MAGFET in the *y*-axis MF; Vo(y,y) is the y-direction OV of the x/y-MAGFET in the *y*-axis MF; and Vo(y,z) is the OV of the z-MAGFET in the *y*-axis MF. As shown in Figure 12, the Vo(y,x) and Vo(y,z) are low in the *y*-axis MF. The slope of the curve Vo(y,x) was 7.8 mV/T, and the slope of the curve Vo(y,z) was 3.2 mV/T. Thereby, the MMS had a cross-sensitivity of 7.8 mV/T (x-direction output) and a cross-sensitivity of 3.2 mV/T (z-direction output) in the *y*-axis MF. The sensitivity of the MMS in the *y*-axis MF was 180 mV/T. Compared to the sensitivity of the MMS, the cross-sensitivity of the MMS in *y*-axis MF was less than 6%.

**Figure 13:**
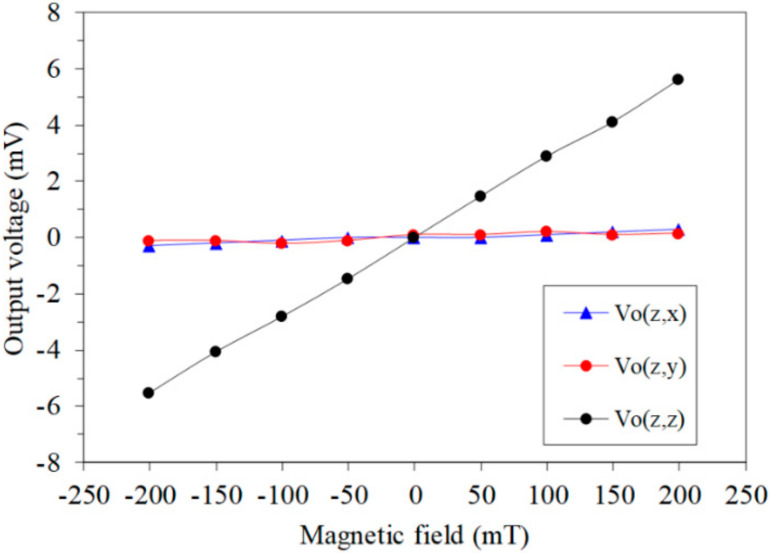
Output of the MMS in the *z*-axis magnetic field.

The *z*-axis MF was applied to the MMS. The gate voltage of the x/y-MAGFET and z-MAGFET was 0.4 V. The bases and collectors of the x/y-MAGFET and z-MAGFET were applied with a bias voltage of 3.3 V. The OV of the x/y-MAGFET and z-MAGFET was recorded using the digital multimeter. Figure 13 shows the OV for each axis of the MMS in the *z*-axis MF, where Vo(z,x) is the x-direction OV of the x/y-MAGFET in the *z*-axis MF; Vo(z,y) is the y-direction OV of the x/y-MAGFET in the *z*-axis MF; and Vo(z,z) is the OV of the z-MAGFET in the *z*-axis MF. As shown in Figure 13, the Vo(z,x) and Vo(z,y) are low in the *z*-axis MF. The slope of the curve Vo(z,x) was 1.3 mV/T, and the slope of the curve Vo(z,y) was 1.1 mV/T. Thereby, the MMS had a cross-sensitivity of 1.3 mV/T (x-direction output) and a cross-sensitivity of 1.1 mV/T (y-direction output) in the *z*-axis MF. The sensitivity of the MMS in the *z*-axis MF was 27.8 mV/T. Compared to the sensitivity of the MMS, the cross-sensitivity of the MMS in *z*-axis MF was less than 6%.

Table 1 lists the sensitivities for various MMS manufactured using CMOS. The MMS developed by Li [16], Xu [30], and Zhao [31] were 1-axis MF sensors, and the MMS presented by Yang [32] was a 2-axis MF sensor. As shown in Table 1, the sensitivity of the MMS in this work in the x- and *y*-axis MF exceeded that of Li [16], Tseng [20], and Kimura [26]. The sensitivity of the MMS presented by Tseng [20] in the *z*-axis MF was lower than that of this work.

## 5. Conclusions

A three-axis magnetic micro sensor with an x/y-MAGFET and a z-MAGFET was manufactured using the commercial 0.18 μm CMOS process. The x/y-MAGFET was used to sense the MF in the *x*- and *y*-axis, and the z-MAGFET was utilized to detect the MF in the *z*-axis. The gates of the x/y-MAGFET and the z-MAGFET increased the mobility of carriers in the surface of the p-substrate and enhanced the sensitivity of the MMS. The MMS was fabricated without a post-CMOS process. The fluxgate MMS presented by Lu [21] needed a post-CMOS process. Fabrication of the MMS was simpler than that of the fluxgate MMS [21]. The experiments showed that the MMS had a sensitivity of 182 mV/T in the *x*-axis MF and a sensitivity of 180 mV/T in the *y*-axis MF. The sensitivity of the MMS was 27.8 mV/T in the *z*-axis MF. The finite element method software, Sentaurus TCAD, was employed to simulate the output voltage of the MMS. The simulated output voltage of the MMS was in agreement with the measured output voltage of the MMS. According to the experimental results, the cross-sensitivity of the MMS in *x*- and *y*-axis MF was less than 6%. The cross-sensitivity of the MMS in *z*-axis MF was also less than 6%. Therefore, the MMS had a high sensitivity and a low cross-sensitivity.

## Figures and Tables

**Table 1:** Sensitivity for various MMS.

MMS	Sensitivity (mV/T)		
	*x*-axis	*y*-axis	*z*-axis
Li [16]	132		
Lin [18]	690	530	91
Tseng [20]	6.5	6.5	0.4
Kimura [26]	140		
Xu [30]			31
Yang [32]	366	365	
Zhao [31]			264
This work	182	180	27.8
